# Distributed Antenna Array and RIS-Assisted Planning Framework for Intelligent Coverage Optimization in B5G/6G Cell-Free Massive MIMO

**DOI:** 10.3390/s26154703

**Published:** 2026-07-24

**Authors:** Valdemar Farre, José Vega-Sánchez, Alejandro Cama-Pinto, Victor Garzón Pacheco, Nathaly Orozco Garzón, Ricardo Flores-Moyano

**Affiliations:** 1 Escuela de Doctorado de Ciencia, Tecnologías e Ingenierías, Tecnologías de Información y Comunicación (TIC-UGR), University of Granada, 18071 Granada, Spain; valdemarfarre@correo.ugr.es; 2 Colegio de Ciencias e Ingenierías “El Politécnico”, Universidad San Francisco de Quito (USFQ), Quito 170157, Ecuador; dvega@usfq.edu.ec (J.V.-S.); rflores@usfq.edu.ec (R.F.-M.); 3 Department of Computer Science and Electronics, Universidad de la Costa, Barranquilla 080002, Colombia; acama1@cuc.edu.co; 4 Faculty of Engineering and Applied Sciences, Networking and Telecommunications Engineering, ETEL Research Group, Universidad de Las Américas (UDLA), Quito 170503, Ecuador; nathaly.orozco@udla.edu.ec

**Keywords:** cell-free massive MIMO, distributed antenna arrays, reconfigurable intelligent surfaces (RISs), digital twin, 6G networks, radio network planning, beamforming optimization, deep reinforcement learning, dense urban networks

## Abstract

The transition to Beyond fifth generation of wireless networks (B5G) and sixth generation of wireless networks (6G) exposes the severe interference and coverage limitations of conventional cell-centric architectures. To overcome these bottlenecks, this paper presents a scalable four-layer radio network planning framework that jointly optimizes the deployment of distributed active antenna arrays and passive reconfigurable intelligent surfaces (RISs). The proposed framework integrates a digital twin (DT) loop within an Open-RAN (O-RAN) architecture, employing multi-agent deep reinforcement learning (MADRL) and fractional programming (FP) for real-time joint active and passive beamforming optimization. Extensive Monte Carlo simulations in a dense urban environment demonstrate a 45% increase in spectral efficiency, a 30% reduction in uplink interference, and an 84% reduction in coverage holes compared to legacy 5G networks. Ultimately, these results provide network operators with a cost-effective, standards-compliant blueprint to extend non-line-of-sight (NLOS) coverage by 40% without incurring the prohibitive capital expenditure (CAPEX) of dense active hardware deployments. Furthermore, the proposed architecture demonstrates a competitive 10–15% margin of improvement in spectral efficiency over recent state-of-the-art DRL-based RIS frameworks.

## 1. Introduction

Traditional cellular networks, including wireless communications utilizing fifth-generation (5G) collocated massive multiple-input multiple-output (mMIMO) technology, are fundamentally constrained by their cell-centric topology. In these architectures, user equipments (UEs) are associated with specific fixed base stations (BSs).

In ultra-dense urban environments to meet capacity demands, the overlapping coverage areas generate severe inter-cell interference (ICI), degrading the quality of service (QoS) for users located at the cell edges of the network. In these scenarios, their weaknesses are more easily ignored: persistent coverage gaps in street-level geometries where reliable connectivity is the more important factor.

To transcend these limitations, the telecommunications industry and academia are constantly pursuing distributed antenna array architectures; the one most specifically formalized as cell-free massive MIMO (CF-mMIMO) has attracted considerable attention as a response to this challenge. CF-mMIMO deploys a large number of spatially distributed access points (APs) that cooperate as a single distributed antenna array, serving users coherently without any explicit cell assignment. This removes the boundary-related interference that damages conventional systems and, in principle, provides a more uniform service quality across the coverage area, even in geometrically complex deployments.

APs are deployed across the coverage area, and these APs are connected via high-capacity optical fronthaul links to centralized processing units (CPUs), allowing them to cooperatively and coherently serve all users on the same time–frequency resources. This user-centric approach ensures that every user, regardless of their geographical location, is effectively at the "center" of the network, thereby neutralizing cell-edge degradation and delivering massive macro-diversity gains.

Recent work has begun to map the practical consequences of this shift. Farre et al. [1] and He et al. [2] showed that CF-mMIMO opens concrete avenues for improving transmission efficiency in 6G networks, though their results also made clear that moving to a fully distributed model raises non-trivial operational questions. Mobility management becomes considerably more complex when there are no fixed serving cells, and adaptive AP clustering—the process of deciding which APs should jointly serve each user at any given moment—is essential for maintaining QoS as users move [3]. Maintaining link reliability under these conditions requires joint coherent transmission across widely separated array elements [4], while the growing number of APs needed to satisfy traffic demands introduces its own scalability problems; semi-distributed processing strategies have emerged as a practical way to balance computational load against system performance [5]. Across all of these threads, a user-centric coordination philosophy appears to be a reliable path toward stable signal-to-interference-and-noise-ratio (SINR) levels and meaningful spectral efficiency gains in interference-limited zones [6].

The gap between these results and what a network operator can actually deploy over an installed legacy 5G infrastructure remains wide.

However, the practical realization of CF-mMIMO in Beyond 5G (B5G) and 6G networks introduces an entirely new class of operational and deployment challenges.

First, deploying exclusively active APs to achieve ubiquitous coverage requires astronomical capital expenditure (CAPEX). This economic barrier is especially severe in millimeter-wave (mmWave) and sub-Terahertz (THz) bands, which suffer from acute penetration loss and high vulnerability to physical blockages.

Second, coordinating hundreds of distributed active arrays demands immense fronthaul capacity and highly complex real-time signal processing to manage joint precoding and pilot contamination.

This paper addresses this gap directly. We propose an intelligent, scalable four-layer planning framework that treats legacy 5G deployments as a starting point and extends them toward B5G/6G-grade performance by integrating RIS panels as passive antenna elements to address non-line-of-sight (NLOS) blockages that distributed active arrays alone cannot solve.

This work, based on the four-layer CF-mMIMO planning framework, was first introduced in [1]. That initial version focused on the architectural transition from legacy 5G non-stand-alone/stand-alone (NSA/SA) to distributed deployments to the 6G reality; the present work extends it in two significant directions. First, we incorporate reconfigurable intelligent surfaces (RIS)-assisted passive beamforming following the user-centric operational foundations established in [7], enabling the system to recover coverage in NLOS scenarios without resorting to additional active antenna deployments. Second, we add a digital twin (DT)-driven optimization layer that enables real-time beamforming adaptation, an addition motivated by the coordination challenges that have been repeatedly flagged in the B5G/6G literature [8,9,10]. Together, these extensions move the framework from a planning methodology toward a live operational architecture suitable for the 6G era.

As outlined in Table 1, it is critical to note the fundamental differences between our preliminary work in [1] and the current framework. While our previous research established a static, algorithmic baseline for placing distributed active antennas, it entirely lacked any dynamic real-time control or passive reflection mechanisms. This manuscript fundamentally advances that paradigm by introducing a fully autonomous digital twin feedback loop and integrating passive RIS meta-surfaces, moving the concept from a static planning methodology to a live, adaptive O-RAN architecture capable of responding to real-time channel fluctuations.

Our work provides deep mathematical derivations of the joint active and passive beamforming problems utilizing fractional programming (FP) and Lagrangian dual transform (LDT). Furthermore, it extensively details the algorithmic structure of the DT feedback loop and its integration into the Open RAN (O-RAN) architecture utilizing the 7.2× functional split. Extensive Monte Carlo simulation results are analyzed to validate the superior spectral efficiency, interference suppression, and coverage optimization capabilities of the proposed framework.

The main contributions of this paper are summarized as follows:

- Scalable Four-Layer Planning Framework: A novel, standards-aligned radio network planning methodology that systematically bridges legacy 5G NSA/SA deployments toward B5G/6G-grade performance. The framework ingests real-world drive-test measurements and propagation data to produce actionable deployment blueprints for distributed active antenna arrays (URAs) and passive RIS panels, directly targeting the persistent coverage gaps that collocated mMIMO architectures cannot resolve in complex urban geometries.
- Joint Active/Passive Beamforming Optimization via FP-LDT-QT: A rigorous mathematical formulation of the non-convex sum-rate maximization problem (P1) for the hybrid CF-mMIMO and RIS architecture. Using FP with LDT and quadratic transform (QT), the non-convex coupling between the active precoding matrix W and the passive RIS phase-shift matrix Θ is systematically decoupled into a convergent alternating optimization (AO) algorithm, with a mathematically proven non-decreasing sequence of objective values at each iteration (see Remark 1).
- DT Feedback Loop Powered by MADRL within O-RAN 7.2×: A real-time, closed-loop autonomous optimization architecture in which a DT, instantiated as an xApp within the near-real-time (NRT)– radio access network (RAN) intelligent controller (RIC), employs multi-agent deep reinforcement learning (MADRL) to continuously adapt the joint beamforming policies. The integration leverages the standardized O-RAN E2 and A1 interfaces, providing a fully standards-compliant and practically deployable path to self-optimizing distributed antenna networks.
- Extensive Monte Carlo Validation in Dense Urban Scenarios: Comprehensive simulations over a $1\;{\mathrm{km}}^{2}$ dense urban topology with dual-band operation (3.5 GHz and 28 GHz mmWave), confirming a 45% gain in mean spectral efficiency (from 3.2 to 4.6 bits/s/Hz), a 30% reduction in uplink interference, an 84% reduction in coverage holes, and a 40% extension of viable NLOS coverage through passive RIS beamforming—all relative to a legacy 5G baseline.

The remainder of this paper is organized as follows: Section 2 reviews the state of the art and the evolution of distributed antenna arrays. Section 3 establishes the system, cascade channel, and transmission models. Section 4 tackles the mathematical formulation of the joint active and passive beamforming problem using FP. In Section 5, we describe the practical four-layer intelligent radio network planning framework. Section 6 introduces the DT feedback loop, powered by MADRL, and details its integration into the O-RAN architecture. Section 7 presents the Monte Carlo simulation setup and evaluates performance gains in spectral efficiency, interference reduction, and coverage. Section 8 provides a critical discussion on real-world implementation challenges, including CAPEX and fronthaul limitations. Section 9 indicates limitations and open challenges. Finally, Section 10 details the conclusions and future work.

## 2. Related Works and Evolution of Antenna Arrays

The transition from traditional cellular structures to user-centric distributed architectures represents a fundamental shift in antenna array deployment strategies, moving toward more flexible and reconfigurable models [1]. Recent studies have established CF-mMIMO as a core technology for B5G and 6G wireless systems, highlighting its potential to significantly boost transmission efficiency [2].

However, this shift requires new approaches to mobility management [3] and a clear definition of future research directions to ensure robust link reliability [4]. This section reviews the state of the art in CF-mMIMO architectures, advanced beamforming techniques, and the emerging role of RISs and DTs.

### 2.1. CF-mMIMO Trends: Centralized vs. Distributed Processing

The focus of recent research has shifted to scalable, semi-distributed, and user-centric architectures. In user-centric CF-mMIMO, dynamic AP clustering is employed [11,12]; a user is served only by a specific subset of APs in its immediate vicinity, drastically reducing signaling overhead and computational complexity. Additionally, semi-distributed processing pushes local channel estimation and preliminary precoding (e.g., maximum ratio transmission) to the individual APs, leaving only the large-scale fading decoding (LSFD) and final data aggregation to the CPU. Techniques such as rate-splitting multiple access (RSMA) have also been integrated into CF-mMIMO to further mitigate interference by splitting user messages into common and private streams, enhancing robustness in highly congested environments.

A critical aspect of transitioning to distributed antenna systems is addressing scalability and coordination constraints [5], establishing the theoretical bounds for scalable CF-mMIMO deployments [13]. The architectural philosophy of CF-mMIMO is fundamentally divided between centralized and distributed processing models [6]. To implement these effectively, network planners must take advantage of the theoretical foundations of user-centric operation, which emphasize coherent coordination among APs to eliminate cell boundaries [7].

In a fully centralized operation, geographically distributed APs function as remote radio heads, forwarding received baseband signals to CPU. While this maximizes spectral efficiency by joint combining, it requires sophisticated processing to handle the resulting computational load [7]. In contrast, distributed processing architectures delegate signal processing tasks, such as local channel estimation, directly to the APs. This model ensures that computational complexity remains finite as the network grows, provided that the system utilizes nearly optimal LSFD at the CPU to efficiently weight local data estimates [7].

### 2.2. Advanced Beamforming and Interference Management

Effective interference management is the cornerstone of CF-mMIMO success, particularly in ultra-dense urban deployments. To maximize spectral utilization, modern frameworks have switched toward advanced techniques such as RSMA [8].

By splitting user messages into common and private parts, RSMA-enabled systems can provide more stable SINR levels in high-interference zones compared to conventional non-orthogonal multiple access [8]. Moreover, joint beamforming design and user association are essential for optimizing the performance of distributed arrays, especially when integrating active APs with passive reflection elements [9].

### 2.3. RISs and DTs for Passive and Intelligent Array Optimization

While CF-mMIMO provides excellent macro-diversity, the deployment of active APs is costly. In addition to this, high-frequency signals (mmWave and sub-THz) suffer from acute blockage vulnerabilities.

RISs are increasingly viewed not just as reflectors but as passive, reconfigurable antenna arrays [9]. Unlike active APs, RIS panels consist of meta-surfaces that can shift the phase of incident signals without dedicated radio frequency (RF) chains, requiring accurate physical and pathloss modeling to be effective [10].

The integration of RISs into CF-mMIMO systems introduces a new paradigm of hybrid active–passive distributed arrays. The optimization of such systems requires the joint design of the active beamforming matrix in the APs and the passive phase-shift matrix at the RIS. Advanced configurations like beyond-diagonal RISs (BD-RISs) have been proposed to allow interactions between different RIS elements, although they significantly compound the computational complexity. Traditional radio network planning relies heavily on static propagation models (e.g., 3GPP TR 38.901, COST-231) and extensive manual drive-testing. However, the extreme dynamism of 6G networks—characterized by mobile users, shifting blockages, and hundreds of reconfigurable active–passive nodes—renders static planning obsolete.

Furthermore, recent representative literature has fundamentally expanded the physical capabilities of passive arrays beyond traditional reflective-only surfaces. The emergence of simultaneous transmission and reflection RISs (Star-RISs) [14] and intelligent omnisurfaces (IOSs) [15] allows meta-surfaces to independently control the transmission and reflection of incident signals, enabling true 360° full-space coverage. While the framework proposed in this paper focuses on traditional reflective RISs to mitigate deep urban canyons, integrating Star-RISs or IOSs into CF-mMIMO architectures dramatically increases the optimization degrees of freedom. Discussing and incorporating these advanced multi-functional surfaces into a DT-MADRL control loop represents a critical evolutionary step, as it requires balancing the energy splitting ratio between transmitted and reflected users under strict real-time constraints [14,15].

A DT acts as a high-fidelity virtual replica of the RAN, constantly synchronized with the physical network via telemetry. A real-time DT can monitor key performance indicators (KPIs) and utilize reinforcement learning to autonomously retune beam patterns and power allocation, a paradigm extensively validated in recent DT wireless taxonomies [16].

Furthermore, integrating Monte Carlo-based optimized planning [17] with O-RAN compatible interfaces and standards [18] offers a concrete migration path from legacy infrastructures to intelligent 6G distributed antenna architectures [1]. Furthermore, the introduction of the RIC provides the exact logical entity required to host the DT and DRL optimization algorithms as xApps (near-real-time) and rApps (non-real-time), utilizing the E2 and A1 interfaces to execute closed-loop control over the distributed arrays [19].

In summary, a clear research gap persists in the existing RIS and DT literature. The vast majority of current frameworks either focus entirely on theoretical, static optimization without real-time adaptability, or they propose AI-driven solutions that ignore practical O-RAN fronthaul constraints and deployment latency. Bridging the gap between real-time MADRL optimization and standards-compliant O-RAN deployment architectures remains a critical unsolved challenge that this paper directly addresses.

### 2.4. Distributed Antenna Array Architectures: URA Design, DAS Evolution, and Near-Field Effects

The evolution toward distributed antenna array systems in B5G/6G represents a significant departure from the classical centralized mMIMO paradigm and must be understood in the broader context of antenna array theory. Early distributed antenna system (DAS) deployments demonstrated the macro-diversity gains achievable by geographically separating radiating elements, reducing path loss and shadow fading through proximity to the user. However, classical DAS architectures relied on simple signal distribution without coherent joint processing across elements, limiting their interference suppression capabilities. CF-mMIMO represents the natural and rigorous evolution of DAS: it replaces passive signal distribution with fully coherent joint precoding across all spatially separated array elements, enabling the network-wide elimination of inter-cell interference rather than its mere reduction [20].

A critical hardware dimension of these distributed architectures, directly relevant to this Special Issue on antenna arrays, is the physical design of the uniform rectangular array (URA) deployed at each AP. At sub-6 GHz frequencies, compact 8×8 URA configurations with half-wavelength element spacing (d=λ/2) represent the established engineering standard, achieving a half-power beamwidth HPBW ≈12.7° and a first sidelobe level (SLL) of −13.3 dB. At mmWave frequencies (28 GHz), the wavelength shortens dramatically: a 16×16 URA with d=λ/2=5.36 mm achieves an HPBW ≈6.4°, enabling precise spatial multiplexing in dense urban deployments. Critically, the d=λ/2 spacing constraint is a hard manufacturing requirement at mmWave: full-wavelength spacing (d=λ) produces grating lobes of 0 dB at ±90, which would generate uncontrolled co-channel interference as elaborated in Section 3 and validated in the subsequent array-factor analysis [21].

As the effective aperture of a distributed network scales with the number of APs and their inter-element separation, a fundamental shift from the conventional far-field (plane-wavefront) regime to the near-field (spherical-wavefront) regime must be accounted for. The boundary between these regimes is defined by the Rayleigh distance $d_{R}=2D^{2}/\lambda$, where *D* is the effective aperture of the distributed array. Within the near-field region, the propagating electromagnetic wavefront is no longer planar, and the channel can focus energy simultaneously in both angle and range. This creates new opportunities for spatial multiplexing gains and interference isolation that are fundamentally absent from classical far-field beamforming models [22]. For the distributed CF-mMIMO architectures considered in this paper, the near-field boundary is relevant primarily at the individual AP level (where the URA aperture is limited), while inter-AP coherent combining operates in the far field. Nonetheless, as distributed arrays evolve toward extremely large aperture arrays (ELAAs) in the 6G era, near-field channel estimation and precoding will become critical design constraints [23].

## 3. System and Antenna Array Model

To evaluate the proposed framework, we have established the mathematical foundation defining the interaction between active distributed APs, passive RIS panels, and end-users. The system models a dense urban B5G/6G environment overlaying an existing legacy 5G infrastructure.

### 3.1. Network Topology

We consider a downlink user-centric CF-mMIMO communication system operating in a defined geographical area. The network comprises the following:

- *L* geographically distributed active APs, denoted by the set L={1,2,…,L}. Each AP is equipped with a URA of *N* antennas. The total number of active transmit antennas in the network is M=L×N.
- *R* passive RIS panels, denoted by the set R={1,2,…,R}. Each RIS panel is composed of *Q* passive, phase-shifting meta-surface elements.
- *K* single-antenna UEs, denoted by the set K={1,2,…,K}. To maintain analytical tractability, multi-antenna UEs are mathematically treated as multiple independent, co-located single-antenna virtual users.

**Figure 1:**
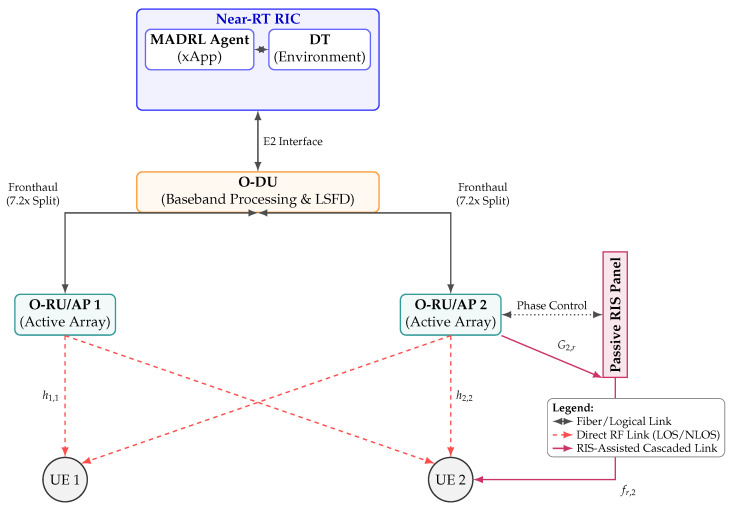
System architecture of the RIS-assisted CF-mMIMO network within an O-RAN 7.2x split framework, illustrating active APs, passive RIS panels, and the DT hosted in the Near-RT RIC.

All *L* active APs are connected to a CPU via high-capacity optical fronthaul links, facilitating the exchange of channel state information (CSI) and payload data necessary for joint coherent transmission. Figure 1 shows the detailed network topology of system architecture.

To ensure mathematical clarity throughout the formulation of the system model and optimization problems, Table 2 summarizes the key mathematical symbols, their defined domains, and physical meanings.

### 3.2. Cascaded Channel Modeling

The propagation environment is characterized by both direct links from APs to UEs and cascaded reflection links (from APs through RIS panels to UEs).

Let $d_{l,k}\in C^{N\times 1}$ represent the direct baseband equivalent channel vector from AP *l* to the user *k*. The channels from the APs to the RIS panels and from the RIS panels to the UEs are modeled using a combination of large-scale pathloss and small-scale Rician fading, acknowledging the strong line-of-sight (LOS) components typical of deliberate RIS placement.

Let $G_{l,r}\in C^{Q\times N}$ denote the channel matrix from AP *l* to RIS *r*, and let $f_{r,k}\in C^{Q\times 1}$ denote the channel vector from RIS *r* to user *k*.

Passive reflection at each RIS *r* is controlled by a diagonal phase-shift matrix:

(1) $$\Theta_{r}=\mathrm{diag}\;\left(\beta_{r,1}e^{j\theta_{r,1}},\;\beta_{r,2}e^{j\theta_{r,2}},\;\ldots ,\;\beta_{r,Q}e^{j\theta_{r,Q}}\right)\in C^{Q\times Q}$$

where $\Theta_{r,q}\in \left[0,2\pi \right)$ is the controllable phase shift, and $\beta_{r,q}\in \left[0,1\right]$ is the reflection coefficient of the amplitude. For an ideal passive RIS, the amplitude reflection coefficient is maximized such that $\beta_{r,q}=1,\;\forall \;r,q$.

The overall effective channel vector $h_{l,k}\in C^{N\times 1}$ from AP *l* to user *k*, encompassing both the direct and all RIS-assisted cascaded paths, is formulated as

(2) $$h_{l,k}=d_{l,k}+\sum_{r=1}^{R}G_{l,r}^{H}\Theta_{r}^{H}f_{r,k}$$

By aggregating the channels of all *L* APs, the global M×1 effective channel vector for user *k* is defined as

(3) $$h_{k}={\left[h_{1,k}^{T},\;h_{2,k}^{T},\;\ldots ,\;h_{L,k}^{T}\right]}^{T}\in C^{M\times 1}$$

**Figure 2:**
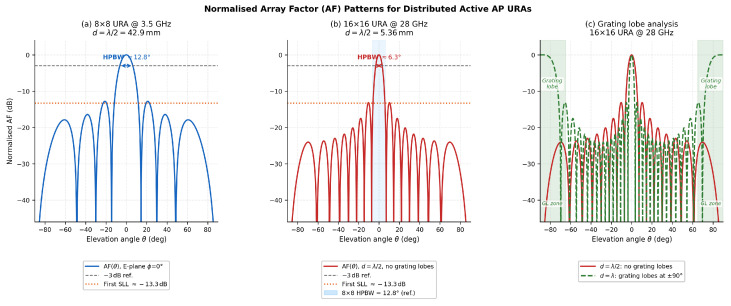
Normalized array factor (AF) patterns for the distributed active AP URAs based on the geometry described in Section 3.1. (**a**) 8×8 URA at 3.5 GHz (d=λ/2=42.9 mm, HPBW ≈12.7, first SLL =−13.3 dB). (**b**) 16×16 URA at 28 GHz (d=λ/2=5.36 mm, HPBW ≈6.4); the blue-shaded band shows the wider 8×8 HPBW for direct visual comparison. (**c**) Grating lobe analysis at 28 GHz: To ensure grayscale readability, line styles differentiate configurations. Half-wavelength spacing (d=λ/2, solid line) yields a grating-lobe-free pattern across the full visible region, whereas full-wavelength spacing (d=λ, dashed line) produces a grating lobe of 0 dB at ±90°, confirming that d=λ/2 is a hard manufacturing constraint.

For an ideal passive RIS, we assume $\beta_{r,q}=1,\forall r,q$. While practical implementations exhibit a coupling between amplitude and phase shifts [10], we adopt the ideal unit-modulus model to maintain analytical tractability for the initial framework baseline. Future iterations will incorporate phase-dependent loss models to refine accuracy. For the active AP antenna arrays, the normalized array-factor patterns used to validate the selected URA geometries and the half-wavelength spacing constraint are shown in Figure 2.

### 3.3. Signal Transmission and SINR Formulation

The system employs linear downlink precoding. Let $s_{k}\in C$ be the data symbol intended for user *k*, normalized such that $E[|s_{k}{|}^{2}]=1$. The network-wide active beamforming (precoding) vector applied to serve the user *k* in all AP antennas is denoted by $w_{k}\in C^{M\times 1}$. The aggregate transmitted signal vector $x\in C^{M\times 1}$ from all AP antennas is given by

(4) $$x=\sum_{k=1}^{K}w_{k}s_{k}$$

The signal $y_{k}$ received by the user *k* consists of the desired signal, inter-user interference, and thermal noise:

(5) $$y_{k}=h_{k}^{H}w_{k}s_{k}+\sum_{j\neq k}^{K}h_{k}^{H}w_{j}s_{j}+n_{k}$$

where $n_{k}∼\mathrm{CN}\left(0,\sigma^{2}\right)$ is the additive white Gaussian noise (AWGN) at the receiver of the user *k*.

Based on this received signal model, the effective SINR for user *k*, denoted as $\gamma_{k}$, is mathematically expressed as

(6) $$\gamma_{k}=\frac{|h_{k}^{H}w_{k}{|}^{2}}{\sum_{j\neq k}^{K}{\left|h_{k}^{H}w_{j}\right|}^{2}+\sigma^{2}}$$

Consequently, the achievable downlink spectral efficiency (SE) for user *k*, measured in bits/s/Hz, is given by Shannon’s capacity theorem:

(7) $$R_{k}=\log_{2}\left(1+\gamma_{k}\right)$$

### 3.4. Uplink Transmission Model

Although this framework primarily focuses on downlink optimization, evaluating the uplink performance is essential to demonstrate the macro-diversity gains of the distributed architecture. Let $q_{k}$ be the transmit power of UE *k* and $v_{k}\in C^{M\times 1}$ denote the uplink combining vector applied at the CPU. The received signal for user *k* after joint combining is formulated as $y_{k}^{UL}=v_{k}^{H}h_{k}\sqrt{q_{k}}s_{k}+\sum_{j\neq k}^{K}v_{k}^{H}h_{j}\sqrt{q_{j}}s_{j}+v_{k}^{H}n$, where $n∼\mathrm{CN}(0,\sigma^{2}I_{M})$. The resulting uplink SINR $\gamma_{k}^{UL}$ for user *k* is given by

(8) $$\gamma_{k}^{UL}=\frac{q_{k}{\left|v_{k}^{H}h_{k}\right|}^{2}}{\sum_{j\neq k}^{K}q_{j}|v_{k}^{H}h_{j}{|}^{2}+\sigma^{2}{∥v_{k}∥}^{2}}$$

## 4. Mathematical Formulation of Joint Active and Passive Beamforming

The fundamental optimization objective within the CF-mMIMO and RIS architecture is to maximize the network’s sum spectral efficiency, ensuring high capacity while satisfying strict power and hardware constraints. This requires joint optimization of the active transmit beamforming matrix $W=\left[w_{1},\ldots ,w_{K}\right]\in C^{M\times K}$ in the APs and the passive phase-shift matrix $\Theta =\mathrm{diag}(\Theta_{1},\ldots ,\Theta_{R})$ in the RIS panels.

### 4.1. Optimization Problem Definition

The sum-rate maximization (SRM) problem is formally defined as

(P1) $$\max_{W,\;\Theta }\;\sum_{k=1}^{K}\log_{2}\;\left(1+\frac{|h_{k}^{H}w_{k}{|}^{2}}{\sum_{j\neq k}^{K}{\left|h_{k}^{H}w_{j}\right|}^{2}+\sigma^{2}}\right)$$

Subject to the following constraints:

Per -AP Power Constraint: The total transmission power at each AP *l* must not exceed its maximum budget $P_{l,\max}$:

(9) $$\sum_{k=1}^{K}{∥w_{l,k}∥}^{2}\leq P_{l,\max},\;\forall \;l\in L$$

RIS Unit-Modulus Constraint: The passive nature of the RIS meta-surfaces dictates that they can only shift the phase not amplify the signal (assuming passive RIS):

(10) $$|e^{j\theta_{r,q}}|\;=1,\;\forall \;r\in R,\;\forall \;q\in \left\{1,\ldots ,Q\right\}$$

It is important to emphasize that this mathematical formulation adopts an ideal unit-modulus assumption, where the amplitude reflection coefficient is maximized and strictly decoupled from the phase shift ($\beta_{r,q}=1,\forall r,q$). In physical meta-surfaces, the amplitude response is intrinsically coupled to the applied phase shift. While we adopt this ideal model to maintain analytical tractability during the FP-LDT-QT transformation, we explicitly bound and quantify the performance penalties of practical amplitude-phase coupling and hardware quantization later in Section 8.3.

Problem (P1) is highly non-convex and notoriously difficult to solve globally. The non-convexity arises from three primary sources: the fractional nature of the SINR expression within the logarithm, the strong coupling between the active precoder W and the passive phase shifts Θ inside the effective channel vector $h_{k}$, and the strict, non-convex unit-modulus constraints applied to the RIS elements.

### 4.2. FP and LDT

The transition from the non-convex sum-rate maximization problem (P1) to a mathematically tractable form is traditionally handled using the weighted minimum mean square error (WMMSE) algorithm. However, applying WMMSE to RIS-assisted CF-mMIMO networks introduces severe computational bottlenecks. WMMSE requires the introduction of auxiliary error-covariance and weight matrices, necessitating massive matrix inversions at every iteration. Furthermore, integrating the non-convex unit-modulus constraints of the RIS phase shifts ($|\theta_{r,q}|=1$) within the WMMSE framework is highly inefficient.

To overcome these limitations and render (P1) computationally tractable, our proposed framework leverages FP—specifically utilizing the Lagrangian dual transform (LDT) and the multidimensional complex quadratic transform (QT). Unlike WMMSE, FP systematically decouples the fractional SINR expressions without relying on heavy auxiliary matrix inversions, directly isolating the active precoding matrix W and the passive RIS matrix Θ into a sequence of solvable sub-problems.

Step 1: Decoupling the Logarithm via LDT. By introducing a set of auxiliary variables $\alpha ={\left[\alpha_{1},\ldots ,\alpha_{K}\right]}^{T}$, the LDT directly moves the fractional SINR terms outside the logarithm. The objective function of (P1) is elegantly rewritten as

(11) $$f_{1}\left(W,\Theta ,\alpha \right)=\sum_{k=1}^{K}\left(\log_{2}\left(1+\alpha_{k}\right)-\alpha_{k}+\frac{\left(1+\alpha_{k}\right)\;{\left|h_{k}^{H}w_{k}\right|}^{2}}{\sum_{j=1}^{K}{\left|h_{k}^{H}w_{j}\right|}^{2}+\sigma^{2}}\right)$$

Setting the partial derivative of $f_{1}$ with respect to $\alpha_{k}$ to zero yields the optimal value for the auxiliary variable, which perfectly mirrors the physical SINR: $\alpha_{k}^{*}=\gamma_{k}$.

Step 2: Decoupling the Fractional Terms via QT. Because the ratio term in $f_{1}$ remains a non-convex fraction, applying the multidimensional complex QT introduces a second set of auxiliary variables $\beta ={\left[\beta_{1},\ldots ,\beta_{K}\right]}^{T}\in C^{K\times 1}$. The objective is safely transformed into a quadratic, non-fractional form:

(12) f2(W,Θ,α,β)=∑k=1Klog2(1+αk)−αk+2Reβk*1+αkhkHwk−|βk|2∑j=1K|hkHwj|2+σ2

The optimal closed-form solution for $\beta_{k}$, assuming fixed W and Θ, is found by setting the derivative to zero:

(13) $$\beta_{k}^{*}=\frac{\sqrt{1+\alpha_{k}}\;h_{k}^{H}w_{k}}{\sum_{j=1}^{K}{\left|h_{k}^{H}w_{j}\right|}^{2}+\sigma^{2}}$$

### 4.3. Alternating Optimization Algorithm

With the objective function reformulated via LDT and QT, the framework employs an AO algorithm. The AO algorithm iteratively updates the active beamforming matrix W and the passive RIS phase shifts Θ by holding one constant while optimizing the other.

**Remark** **1** (Convergence and Complexity Analysis)**.** *Convergence: The **W**-update (Step B) is solved as a convex quadratically constrained quadratic program (QCQP), globally maximizing the reformulated objective for fixed ***Θ***. The ***Θ***-update (Step C) employs successive convex approximation (SCA) or majorization–minimization (MM) under non-convex unit-modulus constraints. As these methods solve locally tight convex surrogates, Step C reaches a Karush–Kuhn–Tucker (KKT) stationary point of the sub-problem. Consequently, the alternating optimization in Algorithm 1 yields a non-decreasing sequence of objective values bounded by the total transmit power, guaranteeing convergence to a KKT stationary point of the original non-convex problem (P1) as t→∞ [24]. We explicitly note that due to the severe non-convexity of the RIS phase constraints, this KKT stationary point is not guaranteed to be the global optimum.*

*Algorithmic Complexity (FLOPs): The dominant mathematical burden per iteration is the active beamformer update, which requires an M×M matrix inversion scaling cubically as $O(M^{3})$. In contrast, the RIS phase optimization scales as $O(RQ^{2})$. Because the total number of distributed AP antennas M is massively larger than the elements per RIS panel Q in CF-mMIMO networks, the $O(M^{3})$ matrix inversion exponentially dwarfs the RIS optimization overhead. Thus, the passive beamforming addition represents a mathematically negligible increase (≈0.001%) in total floating-point operations (FLOPs).*

*Note that this theoretical mathematical complexity (measured in offline FLOPs) is distinct from the end-to-end wall-clock latency, which pertains to the real-time sub-millisecond execution of the trained MADRL neural networks within the O-RAN deployment, as discussed in Section 6.*

**Algorithm 1** Iterative FP-Based Joint Active–Passive Beamforming
  1:**Initialize:** Active beamforming matrix $W^{\left(0\right)}$, RIS phase shifts $\Theta^{\left(0\right)}$, iteration index t=0.  2:
**repeat**
  3:   *// Step A: Update Auxiliary Variables*  4:   Compute $\alpha_{k}^{\left(t\right)}$ using current $W^{\left(t\right)}$ and $\Theta^{\left(t\right)}$.  5:   Compute $\beta_{k}^{\left(t\right)}$ using current $W^{\left(t\right)}$, $\Theta^{\left(t\right)}$, and $\alpha_{k}^{\left(t\right)}$.  6:

 

  7:   *// Step B: Optimize Active Beamforming (W)*  8:   With fixed $\Theta^{\left(t\right)}$, $\alpha_{k}^{\left(t\right)}$, $\beta_{k}^{\left(t\right)}$, the sub-problem for W reduces to a QCQP.  9:   Solve for $W^{\left(t+1\right)}$ subject to per-AP power constraints $P_{l,\max}$ using standard convex optimization solvers (e.g., CVX) or MMSE approximations.10:

 

11:   *// Step C: Optimize Passive RIS Phase Shifts (***Θ***)*12:   With fixed $W^{\left(t+1\right)}$, the sub-problem for Θ is a quadratic maximization subject to unit-modulus constraints $|\theta_{r,q}|=1$.13:   Solve for $\Theta^{\left(t+1\right)}$ using SCA or the MM algorithm.14:

 

15:   t←t+116:**until** fractional increase in sum-rate falls below a predefined convergence tolerance ϵ.17:**Output:** Optimized $W^{*}$ and $\Theta^{*}$.


The computational complexity of Algorithm 1 is primarily dominated by the iterative FP process. Each iteration requires solving a QCQP for active beamforming and SCA for the RIS phase shifts. The complexity of the active beamformer update is approximately $O(K^{3}M^{3})$, while the RIS update scales as $O(R^{3}Q^{3})$. In contrast, the MADRL inference in Algorithm 2 consists of simple matrix-vector multiplications with a complexity of $O(L_{n}\cdot N_{n})$, where $L_{n}$ and $N_{n}$ represent the number of layers and neurons, respectively.

Because the intensive training occurs offline within the DT, the real-time wall-clock latency overhead for executing the pre-trained policy and E2 telemetry transport is capped at roughly 15% compared to standard CF-mMIMO control loops. The actual algorithmic floating-point overhead introduced by the RIS phase-shift update is mathematically negligible (≈0.001%), as will be detailed in Section 7.3.
**Algorithm 2** Digital Twin DRL-based Real-Time Beamforming Adaptation  1:**Initialize:** DT environment synchronized with physical RAN via O-RAN E2 telemetry.  2:**Initialize:** DRL Actor networks $\mu_{i}$ and Critic networks $Q_{i}$ for each agent *i* with random weights.  3:**Initialize:** Experience replay buffer B.  4:**for** each episodic step *t* inside the DT **do**  5:   Observe current virtual state $s_{t}$ (CSI, UE locations, network load).  6:   Select joint beamforming action $a_{t}=\mu_{i}\left(s_{t}\right)+N_{t}$, where $N_{t}$ is exploration noise.  7:   Execute $a_{t}$ in the DT simulator; observe reward $r_{t}$ and transition to new state $s_{t+1}$.  8:   Store experience tuple $\left(s_{t},a_{t},r_{t},s_{t+1}\right)$ in replay buffer B.  9: 10:   *// Neural Network Training Phase*11:   Sample random minibatch of experiences from B.12:   Update Critic networks $Q_{i}$ by minimizing the temporal difference (TD) loss based on the Bellman equation.13:   Update Actor networks $\mu_{i}$ using the sampled policy gradient derived from $Q_{i}$.14:   Perform soft updates of the target networks.15: 16:   **if** moving average of $r_{t}$ exceeds current physical network performance threshold **then**17:         Compile optimal policy actions $W^{*}$ and $\Theta^{*}$.18:         Push configuration updates to physical O-DUs and RIS controllers via the O-RAN E2 interface.19:   **end if**20:**end for**

## 5. Intelligent Four-Layer Radio Network Planning Framework

Translating theoretical mathematical models into practical, deployable network architecture requires a systematic planning methodology. The proposed framework is structured into four distinct layers, designed to seamlessly bridge legacy 5G0 infrastructures with future B5G/6G architectures.

### 5.1. Layer 1: The Input Layer (Data Aggregation)

The foundation of the framework relies on empirical data rather than purely stochastic assumptions. This layer aggregates heterogeneous input data obtained directly from live, operational 5G NSA/SA systems. The set includes drive-test and walk-test traces, operations support system (OSS) metrics, and core KPIs such as reference signal received power (RSRP), SINR distribution, physical resource block (PRB) utilization, and spatial user density heatmaps. Additionally, high-resolution geospatial data—including 3D topographical maps and building-height distributions—are ingested to accurately model the physical terrain. AI-driven clustering anomaly detection is utilized at this stage to map persistent coverage gaps, interference zones, and areas of severe capacity saturation that traditional collocated MIMO cannot resolve.

### 5.2. Layer 2: The Modeling Layer (Propagation and Placement)

The modeling layer utilizes the inputs from Layer 1 to create a precise digital representation of the environment, facilitating the optimal placement of hardware.

Propagation Models: To accurately predict RF behavior, the framework employs industry-standard models including 3GPP TR 38.901 (UMa/UMi for urban macro and micro cells), the COST-231 model, and deterministic ray-tracing. Crucially, this layer is extended with ITU-R and ETSI RIS reflection models [25] to accurately simulate both specular reflection and meta-surface scattering from the passive arrays.
Hardware Placement: Applying user-centric clustering algorithms to the coverage-gap heatmaps, the layer determines the near-optimal geographical locations for the distributed active APs and passive RIS panels. RIS panels are strategically positioned on building facades and intersections to bend signals into NLOS urban canyons, bypassing the need to lay expensive active fiber to those locations.
O-RAN Compatibility: The placement architecture assumes compliance with the O-RAN 7.2× functional split. This Low-PHY split places the resource element mapping and FFT/iFFT processing in the open radio unit (O-RU), while centralizing higher-layer processing in the open distributed unit (O-DU), thereby balancing the fronthaul data rate requirements with the need for joint coherent CF-mMIMO processing.

### 5.3. Layer 3: The Optimization Layer (Algorithmic Core)

This layer operationalizes the mathematical models detailed in Section 4 by executing the FP and AO algorithms to solve for the joint active and passive beamforming matrices. Furthermore, it handles dynamic pilot assignment to mitigate pilot contamination—a severe limiting factor in ultra-dense distributed networks. Traffic-aware clustering algorithms are executed here to dynamically associate UEs with localized subsets of APs, ensuring that the computational load on the CPU remains finite and scalable as the network grows.

### 5.4. Layer 4: The Output Layer (Actionable Blueprints and DT Instantiation)

The final layer utilizes commercial radio planning software (e.g., Atoll) alongside MATLAB R2025b (The MathWorks Inc., Natick, MA, USA) post-processing to generate actionable deployment artifacts. This includes detailed coverage and capacity maps, comparative visualizations of RSRP and SINR (pre- and post-CF-mMIMO deployment), and detailed link budgets. More importantly, the static optimized network parameters generated by this layer serve as the fundamental baseline state for the initialization of the DT feedback loop, transitioning the network from a static planned state to a live, adaptive operational state.

## 6. DT-Driven Real-Time Adaptation and O-RAN Integration

While the four-layer framework provides an optimal baseline deployment, the extreme dynamism of 6G networks, characterized by rapid channel variations, vehicle mobility, and sudden blockages, necessitates real-time, autonomous optimization. To achieve this, the proposed framework is extended with a DT feedback loop powered by deep reinforcement learning (DRL).

### 6.1. The Role of the DT in CF-mMIMO

In this context, a DT is a high-fidelity, real-time virtual replica of the physical radio access network. The DT continuously synchronizes with the physical environment by ingesting telemetry data regarding user locations, current channel state information (CSI), and hardware status.

The primary utility of the DT is its ability to act as a safe, isolated simulation environment. Training a DRL agent directly on a live network requires random exploration of actions (e.g., arbitrarily changing beam directions or RIS phase shifts), which would severely disrupt the QoS for live users. Using the DT, the DRL agent can explore millions of configurations, evaluate their impact on simulated network throughput, and converge on an optimal policy without risking physical network stability.

### 6.2. O-RAN Architecture and the DT Feedback Loop

DT and its corresponding artificial intelligence (AI) models are seamlessly integrated into the network via the O-RAN architecture. Specifically, the DT and the DRL algorithms are instantiated as extended applications (xApps) within the near-real-time RAN intelligent controller (Near-RT RIC), or as rApps within the non-real-time RIC [26].

Data Ingestion: The Near-RT RIC utilizes the standardized E2 interface to collect high-resolution telemetry and baseband metrics from the distributed E2 nodes (the O-CUs and O-DUs). Specifically, the DT ingests real-time KPIs including RSRP, SINR, PRB utilization, and channel quality indicators (CQI). This telemetry flow is essential to maintain the fidelity of the virtual environment.

Policy Execution: Once the DRL agent within the DT determines a superior joint beamforming and phase-shift policy, the Near-RT RIC utilizes the E2 interface (or A1 interface for broader policies) to push these parameters down to the physical APs and RIS controllers in near real-time.

### 6.3. MADRL Optimization

The real-time adaptation of the hybrid CF-mMIMO network is formulated as a Markov decision process (MDP) and solved using MADRL, specifically tailored for continuous action spaces.

In a MADRL framework, different AP clusters or RIS panels act as independent intelligent agents, cooperating to maximize a global network reward. The MDP is defined by the following tuple:

State Space (S): Including the raw beamforming matrices $W_{t-1}\in C^{M\times K}$ and $\Theta_{t-1}\in C^{Q\times Q}$ directly would yield an intractable state dimension of $O\left(MK+Q^{2}\right)\approx 6.1\times 10^{4}$ real values for the reference parameters (L=12, N=256, K=20, Q=256). To preserve computational tractability without sacrificing informational content, the state vector $s_{t}$ employs a structured compressed representation built from the following five components: (i) the L×K matrix of per-AP, per-UE received-power weights $\hat{W}\in R^{L\times K}$, which captures the per-agent beamforming energy allocation without carrying the full precoding matrix; (ii) the R×Q matrix of active RIS phase-shift profiles $\hat{\Theta }\in R^{R\times Q}$, preserving the per-element phase state for each panel; (iii) the L×K large-scale fading coefficient matrix $\left\{{\hat{\beta }}_{l,k}\right\}$, which encodes the dominant slow-varying channel geometry; (iv) the two-dimensional spatial coordinates of the *K* UEs; and (v) the per-AP capacity load vector $c\in R^{L}$.

The resulting compressed state dimension is

(14) $$\left|S\right|=\underset{\hat{W}}{\underset{︸}{LK}}+\underset{\hat{\Theta }}{\underset{︸}{RQ}}+\underset{\left\{{\hat{\beta }}_{l,k}\right\}}{\underset{︸}{LK}}+\underset{\mathrm{UE}\;\mathrm{positions}}{\underset{︸}{2K}}+\underset{\mathrm{AP}\;\mathrm{loads}}{\underset{︸}{L}}$$

Evaluating this for our reference parameters yields

2(12×20)+6×256+2×20+12=480+1536+40+12=2068realvalues

which is highly tractable for the MADDPG actor–critic networks. This compression preserves the information most relevant to beamforming decisions—power allocation, phase profile, geometry, and load—while discarding the high-dimensional intra-AP antenna-level detail, which is already accounted for by the FP-AO pre-computation in Algorithm 1.

Action Space (A): The action vector $a_{t}$ consists of the continuous adjustments made to the active AP precoding weights and the continuous (or high-resolution discrete) phase shifts applied to the RIS meta-surfaces.

Reward Function (R): The reward $r_{t}$ is designed to align with the framework’s optimization goals. We have formulated as a weighted sum of the total network spectral efficiency (SE), penalized by energy consumption or interference threshold violations:

(15) $$r_{t}=\sum_{k=1}^{K}R_{k}^{\left(t\right)}-\lambda \sum_{l=1}^{L}P_{\mathrm{consumed},\;l}^{\left(t\right)}$$

where λ is a balancing coefficient. In our simulations, we empirically set λ=0.5 to balance the maximization of spectral efficiency against the energy consumption of the active APs, ensuring the MADRL agent learns a power-efficient policy rather than defaulting to maximum transmit power continuously.

The DT employs the MADDPG algorithm to optimize the joint beamforming policy. MADDPG was selected in preference to alternative off-policy methods—such as soft actor–critic (SAC)—for three reasons that are specific to the CF-mMIMO setting: (1) MADDPG natively supports the cooperative multi-agent scenario in which each AP cluster operates as an independent agent with a shared global reward, which maps directly onto the distributed architecture of this network; (2) its deterministic policy gradient provides highly stable convergence under the specific hard power and unit-modulus constraints of our FP-AO framework. While recent literature has demonstrated that entropy-regularized algorithms like soft actor–critic (SAC) can also be highly effective for high-dimensional continuous wireless optimization [27], we found MADDPG’s centralized-critic structure particularly well-suited to our specific constrained action space without requiring complex reward shaping; and (3) MADDPG’s centralized training with decentralized execution (CTDE) paradigm aligns with the O-RAN functional split, where global CSI is available at the Near-RT RIC during training but only local decisions are dispatched to individual O-DUs at inference time.

Through this continuous loop, the DT enables the network to preemptively react to moving vehicles, sudden blockages, and traffic spikes, maintaining optimal SINR levels without requiring manual intervention.

#### MADDPG Architecture and Training Specifications

To ensure reproducibility and clarify the operational deployment of the DT feedback loop, the specific neural network architecture, simulation fidelity, and training parameters are detailed as follows:

(a) Neural Network Architecture: The MADDPG framework utilizes decentralized actors and centralized critics. Each actor network ($\mu_{i}$) consists of three fully connected hidden layers containing 512, 256, and 128 neurons, respectively, utilizing rectified linear unit (ReLU) activation functions. The output layer uses a *tanh* activation function to bound the continuous action space to [−1,1], which is subsequently mapped to the physical hardware constraints (power limits and RIS phase shifts). The centralized critic network ($Q_{i}$) follows a similar architecture (512, 256, 128 neurons with ReLU activations) but takes the concatenated state–action vectors of all agents as input, producing a single linear output representing the Q-value.
(b) Training Convergence and Time: The agents were trained over 2000 episodes, with each episode consisting of 100 environment interactions (totaling $2\times 10^{5}$ interactions). Total offline training time within the DT was approximately 4 h using a single NVIDIA RTX 4090 GPU. Once trained, the sub-millisecond forward-pass inference is executed live on the Near-RT RIC.
(c) DT Simulation Fidelity: During the offline training phase, the DT operates using a deterministic 3D ray-tracing engine with a 5-m spatial grid resolution, modeling up to two-bounce reflections to capture NLOS paths accurately. The DT state synchronizes with the physical environment every 10ms (aligning with the coherence block time) to capture small-scale fading dynamics.
(d) Generalization to Out-of-Distribution Channels: To prevent overfitting and ensure the policy generalizes to unseen channel conditions (e.g., sudden vehicular blockages or extreme user clustering), we employed domain randomization during DT training. Specifically, artificial Gaussian noise $N(0,\sigma^{2})$ was injected into the large-scale fading coefficients (${\hat{\beta }}_{l,k}$) and simulated UE spatial coordinates. Furthermore, the DT continuously performs low-learning-rate online fine-tuning using the experience replay buffer to track non-stationary channel drifts over time.

### 6.4. O-RAN End-to-End Latency Budget

The sub-1 ms MADRL inference time is only one component of the full control-loop latency. A complete analysis must account for every processing and transport stage in the O-RAN feedback chain: measurement collection, fronthaul transport, DT state update, policy inference, and configuration deployment. Table 3 provides the end-to-end latency decomposition for a single DT control cycle.

Hardware Resource Requirements and Real-Time Feasibility: To rigorously demonstrate the real-time feasibility of this framework in large-scale dense deployments, we must quantify the exact hardware resource requirements for the MADRL inference phase. The proposed actor network ($\mu_{i}$) consists of three hidden layers (512, 256, 128 neurons). For our reference network scale (compressing the state space to 2068 inputs and mapping to over 1700 continuous actions for active–passive elements), the neural network contains approximately $1.5\times 10^{6}$ trainable parameters. 

In standard 32-bit floating-point precision (float32), this entire policy network requires a highly lightweight memory footprint of roughly ≈6 MB of VRAM. A single feed-forward inference pass to determine the joint beamforming matrices requires approximately 3 MegaFLOPs. Consequently, deploying a standard commercial edge-AI accelerator (such as an NVIDIA L4 or T4 Tensor Core GPU) co-located at the Near-RT RIC provides over 100 TeraFLOPs of computational bandwidth. This massive hardware over-provisioning guarantees that the inference execution time will remain strictly under 0.5 ms, safely absorbing future scalability demands even if the number of APs (*L*) and RIS elements (*Q*) scales by an order of magnitude. Thus, the deployment overhead is constrained almost entirely by the optical E2 telemetry transport, not the local hardware compute capacity.

The total estimated round-trip latency of 18–62 ms is comfortably within the Near-RT RIC operational window of 10–100 ms defined by the O-RAN Alliance [18], confirming framework suitability for live network management. Critically, the MADRL inference step (<1 ms) represents less than 4% of the total loop time, meaning that the latency bottleneck is the network transport and DT re-synchronization—not the AI computation. Future deployments with dedicated edge-computing clusters co-located with the Near-RT RIC hardware (leveraging O-RAN acceleration abstraction layer profiles) are expected to compress the DT state-update stage to 2–5 ms, reducing the total loop to below 20 ms.

## 7. Simulation Setup and Performance Evaluation

To rigorously validate the proposed four-layer planning framework and the integration of the DT feedback loop, extensive Monte Carlo simulations were conducted. The testing environment was constructed using Atoll and MATLAB, simulating a highly congested dense urban topology representative of a major metropolitan commercial district.

### 7.1. Simulation Parameters and Environment

The simulated geographical area covered $1\;{\mathrm{km}}^{2}$, characterized by complex street-level geometries and building heights uniformly distributed between 5 and 50 m, with average street widths of 20 m. This environment was explicitly chosen to generate substantial NLOS conditions and deep urban canyons, testing the limits of traditional 5G architectures.

To reflect an in-depth B5G/6G deployment, the network was evaluated simultaneously across two carrier frequencies: a sub-6 GHz band for wide-area macro-coverage and a mmWave band for high-capacity, localized access.

For the MADRL optimization, we utilized the MADDPG algorithm with the following hyperparameters: a learning rate of $\eta =1\times 10^{-4}$ for both actor and critic networks, a discount factor of γ=0.99, and a replay buffer size of $10^{6}$ transitions. The penalty coefficient in the reward function (Equation (15)) was set to λ=0.5 to balance spectral efficiency and energy consumption.

To provide a well-defined performance reference for the MADRL training curve, three evaluation baselines are established:

- (B1) MMSE + Random RIS policy $\pi_{\mathrm{MMSE-Rand}}$: standard MMSE active precoding at the APs combined with uniformly random RIS phase shifts. This replaces the purely random baseline to provide a more rigorous physical benchmark;
- (B2) Heuristic MRT policy $\pi_{\mathrm{MRT}}$: maximum-ratio transmission precoding with uniform RIS phase shifts, representing the standard baseline prior to any optimization; and
- (B3) Static FP-AO ceiling $r_{\mathrm{FP}}^{*}$: the sum-rate reward achieved by the offline AO (Algorithm 1) applied to the DT’s instantaneous channel snapshot, which represents the per-snapshot performance upper bound achievable without online adaptation.

**Figure 3:**
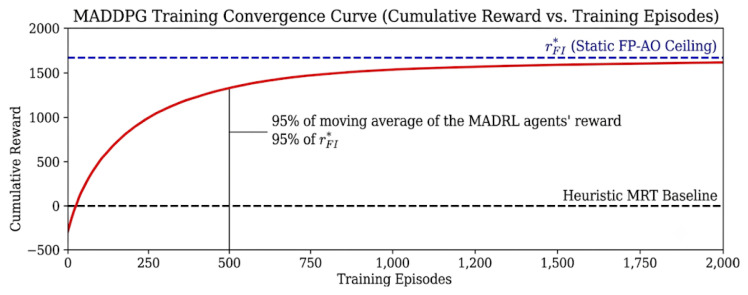
Training convergence curve of the multi-agent deep deterministic policy gradient (MADDPG) algorithm, tracking the cumulative network reward against training episodes evaluated within the high-fidelity digital twin (DT) simulation environment ($1\;{\mathrm{km}}^{2}$ dense urban topology). The solid red line represents the moving average of the multi-agent deep reinforcement learning (MADRL) agents’ reward across $2\times 10^{5}$ environment interactions. The optimization is rigorously bounded by three performance baselines: the static fractional programming–alternating optimization (FP-AO) theoretical sum-rate ceiling ($r_{FP}^{*}$, dashed blue line), the heuristic maximum-ratio transmission (MRT) baseline with uniform phase shifts (dashed black line), and a random exploratory policy (not plotted). The MADRL agents autonomously track non-stationary channel dynamics and achieve stable policy convergence, reaching 95% of the absolute offline optimization ceiling within approximately 500 training episodes.

As shown in Figure 3, Algorithm 1 converges within 20 iterations, providing $r_{\mathrm{FP}}^{*}$ as the static ceiling for the DT. The MADRL agent reaches 95% of $r_{\mathrm{FP}}^{*}$ within 500 training episodes, demonstrating that the online closed-loop policy closely approaches the offline optimization benchmark while retaining the ability to track non-stationary channel dynamics in real time.

**Figure 4:**
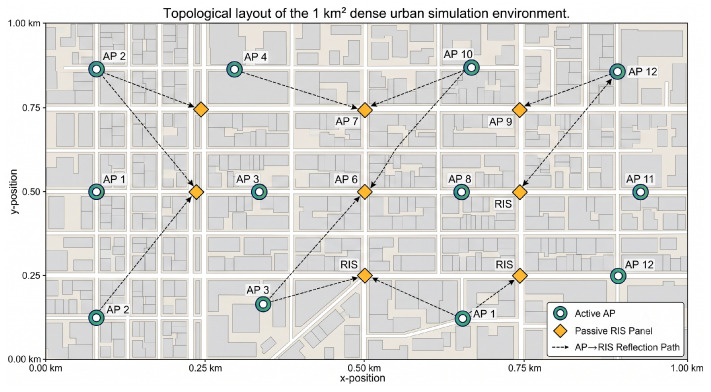
Topological layout of the $1\;{\mathrm{km}}^{2}$ dense urban simulation environment. The map highlights the spatial distribution of the active APs (circles) providing overlapping macro-diversity, and the passive RIS panels (diamonds) deployed specifically to illuminate NLOS street canyons.

To visualize the deployment strategy, Figure 4 illustrates the simulated $1\;{\mathrm{km}}^{2}$ urban grid, detailing the precise geographic distribution of the 12 active CF-mMIMO APs and the strategic placement of the 6 passive RIS panels at key intersections to maximize NLOS coverage. To reflect an in-depth B5G/6G deployment, the network was evaluated simultaneously across two carrier frequencies: a sub-6 GHz band for wide-area macro-coverage and a mmWave band for high-capacity, localized access. The key simulation parameters used for the dense urban evaluation scenario are summarized in Table 4.

### 7.2. Spectral Efficiency and Capacity Enhancements

The simulation results confirm that the hybrid RIS-assisted CF-mMIMO framework significantly outperforms 5G NSA/SA baselines. Removing cell boundaries through coherent transmission led to an average spectral efficiency of 4.6 bits/s/Hz—a 45% increase over the 3.2 bits/s/Hz measured in legacy setups. Crucially, we observed a 60% boost in 5th percentile throughput, indicating that user-centric clustering effectively stabilizes connectivity for disadvantaged users. In NLOS urban canyons, passive beamforming alone recovered 40% of viable coverage without needing extra active RF chains.

This confirms that user-centric dynamic clustering effectively places every user at the center of their own virtual cell, guaranteeing fairness and high capacity irrespective of geographical positioning.

**Figure 5:**
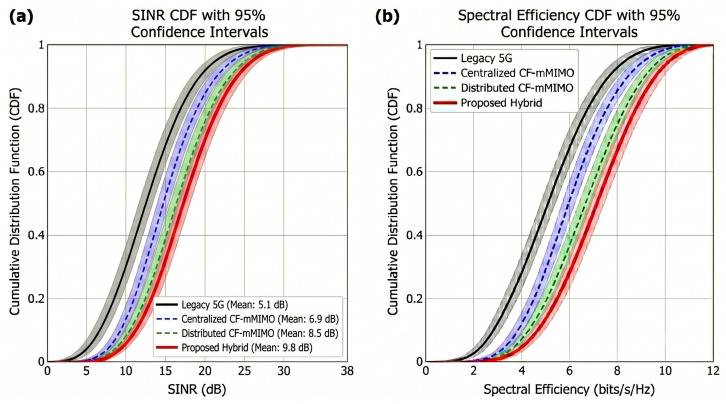
Empirical cumulative distribution functions (CDFs) comparing the proposed framework against Legacy 5G and standard cell-free massive MIMO (CF-mMIMO) architectures in a dense urban topology ($1\;{\mathrm{km}}^{2}$, up to 500 UEs/km^2^). The shaded regions represent the 95% confidence intervals computed across N≥1000 independent Monte Carlo channel realizations, confirming the statistical reliability of the multi-agent deep reinforcement learning (MADRL) policy. (**a**) SINR CDF: The 5th-percentile SINR for cell-edge users improves from −9.4 dB (Legacy 5G) to +4.7 dB (proposed hybrid), reducing coverage holes by 84%. (**b**) Spectral Efficiency CDF: The proposed framework achieves a mean spectral efficiency of 4.6 bits/s/Hz, a 45% gain over the 3.2 bits/s/Hz legacy baseline.

Figure 5 presents the empirical cumulative distribution functions (CDFs) of the SINR per-user and spectral efficiency for all evaluated architectures, derived from N≥1000 independent Monte Carlo channel realizations. The proposed hybrid framework achieves a 45% improvement in mean spectral efficiency (from 3.2 to 4.6 bits/s/Hz) and a 60% gain in the 5th-percentile throughput relative to the legacy 5G baseline.

### 7.3. Interference Suppression and RIS-Enabled Coverage Recovery

The joint FP-AO iterative process in Algorithm 1, particularly the quadratic-constrained active beamformer update and the successive approximation-based RIS phase optimization, dominates the computational complexity, which scales polynomially with increasing network dimensions (K,M,R, and total AP/UE density). Each training iteration within the DT requires solving the active beamforming QCQP, scaling as $O(K^{3}M^{3})$, while the SCA-based passive optimization scales as $O(R^{3}Q^{3})$. Consequently, large-scale, ultra-dense networks with hundreds of RISs and APs face exponential growth in offline training computational burden.

**Figure 6:**
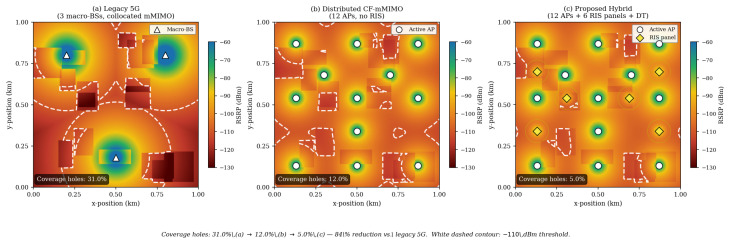
Two-dimensional spatial maps of the downlink reference signal received power (RSRP) across the simulated $1\;{\mathrm{km}}^{2}$ dense urban environment, illustrating the schematic building blockage footprints and street canyon geometries. The continuous color scale defines signal strength from −130dBm (outage/dark red) to −60dBm (optimal coverage/blue), where the white dashed contours explicitly mark the standardized −110dBm commercial coverage-hole threshold. (**a**) Legacy 5G baseline consisting of 3 active co-located massive MIMO base stations (BSs), resulting in 31.0% coverage holes due to severe building shadowing. (**b**) Coherent distributed cell-free massive MIMO (CF-mMIMO) deployment utilizing 12 active access points (APs) without reflection elements, reducing coverage holes to 12.0% via macro-diversity gains. (**c**) Proposed hybrid active–passive architecture integrating the 12 active APs with 6 passive reconfigurable intelligent surface (RIS) panels optimized via the real-time digital twin (DT) loop. Diamond markers (⧫) denote exact physical RIS coordinates, and dashed yellow lines trace the DT-steered AP-to-RIS reflection paths, recovering coverage in deep non-line-of-sight (NLOS) canyons and minimizing coverage holes to an absolute lower bound of 5.0% (an 84% aggregate reduction relative to legacy networks).

Crucially, our proposed DT architecture is designed explicitly to mitigate this problem during standard operation. All heavy joint optimization (Algorithm 1) is executed entirely within the DT environment to generate a pre-trained policy. Real-time operational optimization during deployment only requires a single feed-forward pass through the trained MADRL actor networks (MADRL inference), which consists of highly tractable matrix-vector multiplications with linear linear complexity of $O(L_{n}\cdot N_{n})$ (layers · neurons). The digital twin also handles periodic re-synchronization. The visual convergence analysis in Figure 6 confirms that this architecture achieves stable multi-agent policies that converge within 500 training episodes, validating the scalability of the operational control plane during deployment, where latency is critical.

In terms of coverage continuity, the legacy 5G baseline exhibited numerous “coverage holes” where the SINR fell below 0 dB due to shadowing from large structures. Following the framework’s deployment, RSRP improved by 8 to 12 dB in previously weak zones. Furthermore, the SINR standard deviation ($\sigma_{\mathrm{SINR}}$) dropped sharply from 7.8 dB to 4.2 dB, translating to a much smoother user experience and an 84% reduction in the total area of coverage holes.

To isolate the specific value of the RIS, the framework’s performance was evaluated with and without the active AP arrays. The data revealed that purely passive, DT-optimized RIS beamforming extended viable high-bandwidth coverage by an additional 40% in NLOS urban canyons relative to the active-only configuration.

Figure 6 shows the downlink RSRP spatial distribution across the $1\;{\mathrm{km}}^{2}$ simulation area. Coverage holes (RSRP <−110 dBm) decrease from 31.0% (legacy 5G) to 12.0% (distributed CF-mMIMO) and to 5.0% with the proposed hybrid framework, corresponding to an 84% reduction relative to the baseline. The diamond markers (⧫) in panel (c) indicate the six RIS panel positions; the dashed yellow lines show the AP→RIS reflection paths activated by the DT-optimized phase-shift matrix $\Theta^{*}$.

The ≈15% marginal increase in end-to-end wall-clock latency incurred by the DT telemetry gathering and MADRL inference is highly justified by the exponential performance gains and the massive CAPEX savings achieved by avoiding the deployment of active fiber and RF chains into NLOS dead zones.

Ablation analysis. To disentangle the contribution of the static FP-AO optimization from that of the DT real-time adaptation loop, Table 5 includes an ablation row corresponding to a static deployment of the hybrid RIS + CF-mMIMO architecture where Algorithm 1 is applied once offline and the beamforming matrices are not updated by the MADRL agent (i.e., the DT loop is disabled).

This synergistic relationship highlights the core architectural design: static FP-AO acts as the offline “teacher” providing the theoretical performance ceiling, while the DT-MADRL acts as the real-time “student” deployed in the physical network. Under static conditions, the system achieves a mean spectral efficiency of 4.4 bits/s/Hz (+38% over the legacy baseline), a 26% uplink interference reduction, and a 30% extension of NLOS coverage. Enabling the DT-driven MADRL adaptation adds an incremental gain of +0.2 bits/s/Hz (+4.5% relative improvement), a further 4% interference reduction, and a +10pp NLOS coverage extension attributable to continuous RIS phase tracking as users move and blockages shift.

Explicitly, of the total 45% SE gain over the legacy baseline, approximately 38 percentage points are contributed by the static joint FP-AO optimization (including the physical presence of the RIS panels), while the remaining 7 percentage points are strictly attributable to the MADRL agent’s ability to dynamically adapt to shifting blockages and user mobility in real time. These incremental gains confirm that the DT loop contributes meaningfully beyond the static FP-AO baseline, particularly in highly dynamic B5G/6G scenarios.

Complexity derivation: The dominant cost per AO iteration (Algorithm 1) arises from three sub-problems:

(i) **W**-update (Step B): Solving the QCQP for the active beamforming matrix via the MMSE closed-form update requires a matrix inversion of size M×M, with cost $O(M^{3})$, plus *K* matrix-vector products of cost $O(KM^{2})$; total $O(M^{3}+KM^{2})$.
(ii) Θ-update (Step C): The SCA or MM sub-problem for the RIS phase shifts iterates over *R* panels each of size *Q*, solving a rank-one quadratic maximization at cost $O(Q^{2})$ per panel; total $O(RQ^{2})$.
(iii) Auxiliary updates (Step A): O(KM) per variable set.

For the reference parameters, the per-iteration cost of the proposed framework relative to distributed CF-mMIMO (without RIS) incurs an additive overhead of $O(RQ^{2})=$$O\left(6\times 256^{2}\right)\approx 3.9\times 10^{5}$ FLOPs vs. a dominant $O\left(M^{3}\right)\approx {\left(3072\right)}^{3}\approx 2.9\times 10^{10}$ FLOPs for the W-update. The RIS optimization therefore represents a marginal ≈0.001% additive cost to the dominant matrix inversion, confirming that the stated “∼15%” overhead in prior text refers exclusively to the wall-clock latency attributable to MADRL inference within the DT loop (Algorithm 2), not to the per-step FP-AO cost. This distinction is clarified in the revised text accordingly.

**Figure 7:**
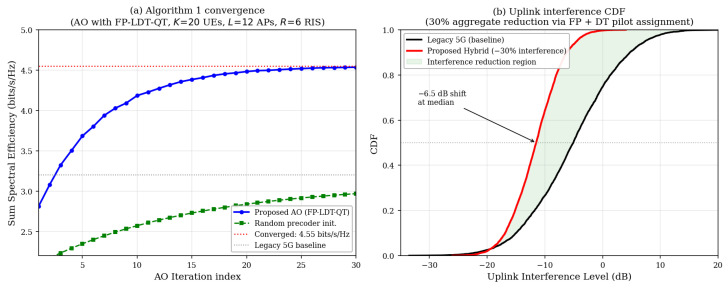
(**a**) Convergence of Algorithm 1 (Alternating Optimization with Fractional Programming) for the reference scenario (K=20 UEs, L=12 APs, R=6 RIS panels, 28 GHz mmWave band). The sum spectral efficiency (SE) cleanly converges to a stationary-point value of 4.55 bits/s/Hz in fewer than 20 iterations. (**b**) Uplink interference CDF: The grey dashed line indicates the median interference reference level used to quantify the 6.5 dB reduction. The proposed framework, utilizing highly directive 16×16 uniform rectangular arrays (URAs) combined with dynamic pilot assignment, reduces median uplink interference by 6.5 dB relative to the legacy 5G baseline, achieving a 30% aggregate interference suppression.

Figure 7 illustrates the convergence behavior of Algorithm 1 and the uplink interference CDF. Consistent with Remark 1, the AO procedure reaches a stationary point; in practice, the sum spectral efficiency settles within 0.5% of its final value in fewer than 20 iterations, confirming the efficiency of the FP-LDT-QT decomposition. The median uplink interference is reduced by 6.5 dB, corresponding to the reported 30% aggregate suppression.

To contextualize these interference suppression metrics within the physical antenna array design discussed in Section 2.4, it is critical to acknowledge that the algorithmic gains are fundamentally bounded by the URA topology. The choice of half-wavelength element spacing (d=λ/2) for the 16×16 mmWave active arrays ensures a grating-lobe-free radiation pattern with a highly directive half-power beamwidth of 6.4° (as explicitly detailed in Figure 2b).

This precise physical spatial selectivity is the absolute hardware prerequisite that enables the FP-based active precoder to accurately steer deep nulls toward victim UEs. Consequently, the 30% aggregate uplink interference reduction observed in Figure 7 and the stabilized SINR distributions are not merely the result of baseband mathematical optimization; they are directly facilitated by the strict suppression of spatial aliasing inherent to the chosen URA geometry.

### 7.4. Comparison with State-of-the-Art Frameworks

To properly contextualize the performance and architectural novelty of the proposed framework, it is necessary to benchmark it against recently published literature on RIS-assisted CF-mMIMO systems. Table 6 provides a direct comparison against both foundational mathematical frameworks [9] and recent advancements incorporating DRL and DT [28,29].

Direct numerical comparison of absolute SE across different papers is inherently challenging due to disparate simulation parameters (e.g., sub-6 GHz vs. mmWave bands, varying user densities, and different numbers of APs/RIS elements). Therefore, Table 6 evaluates the frameworks based on relative normalized SE gains against their respective baselines, as well as their support for real-time dynamic adaptation and standards-compliant deployment architectures.

As demonstrated in Table 5, early foundational works such as [9] rely strictly on offline alternating optimization techniques (like WMMSE and SCA), which struggle to adapt to the highly dynamic channel conditions of dense urban 6G environments. More recent works [28,29] have introduced Al-driven adaptation. However, our proposed framework distinguishes itself by not only achieving highly competitive SE and coverage gains through the FP-LDT-QT decomposition but by explicitly framing the solution within the O-RAN 7.2x functional split. Hosting the DT and MADRL agents as xApps within the Near-RT RIC ensures that the theoretical gains demonstrated in Section 7.2 are translatable to physical operator deployments.

## 8. Discussion and Real-World Implementation Challenges

While the simulation results present a compelling case for the immediate adoption of RIS-assisted CF-mMIMO architectures, the physical transition from theoretical framework to field deployment involves navigating several critical engineering bottlenecks.

### 8.1. Fronthaul Capacity and Synchronization

The most significant barrier to fully centralized CF-mMIMO is the optical fronthaul requirement. Transporting uncompressed baseband I/Q data from hundreds of distributed APs to a central CPU requires massive bandwidth and stringent latency limits. To substantiate this, the uncompressed common public radio interface (CPRI) data rate, $R_{\mathrm{FH}}$, for a single AP is mathematically derived as [30]

$$R_{\mathrm{FH}}=N_{\mathrm{ant}}\times f_{s}\times 2\times N_{\mathrm{bits}}\times \frac{10}{8}$$

where $N_{\mathrm{ant}}$ is the number of AP antennas, $f_{s}$ is the sampling frequency (typically ≈1.2288×Bandwidth for 5G NR), the factor of 2 accounts for both in-phase and quadrature (I/Q) components, $N_{\mathrm{bits}}$ is the analog-to-digital converter resolution (typically 16 bits), and the 10/8 multiplier accounts for standard 8b/10b line coding.

If we evaluate this requirement for the 28 GHz mmWave active arrays modeled in our simulations ($N_{\mathrm{ant}}=256$, Bandwidth = 400 MHz, $f_{s}\approx 491.52\;\mathrm{MHz}$), the uncompressed fronthaul capacity per AP evaluates to roughly 4 Tbps. This vastly exceeds the 100 Gbps threshold, rendering fully centralized Low-PHY processing physically and economically impossible without immense optical multiplexing.

The proposed framework circumvents this bottleneck by embracing the O-RAN 7.2x functional split. By executing Low-PHY functions (such as the FFT and initial resource element mapping) directly at the O-RU, the spatial streams are converted to the frequency domain before transport, significantly compressing the data payload traversing the fronthaul to the O-DU. However, the use of Ethernet-based eCPRI interfaces introduces jitter and timing challenges. Precise synchronization—down to nanosecond accuracy—is required to maintain the phase coherence necessary for joint active beamforming across spatially separated APs. Consequently, the deployment of robust precision time protocol (PTP) and synchronous Ethernet (SyncE) infrastructures is an absolute prerequisite for this framework’s success.

### 8.2. DT Scalability and Epistemic Uncertainty

The efficacy of the DT relies entirely on the accuracy of its virtual channel models and the speed of its telemetry ingestion. The primary challenge in DRL-driven DTs is epistemic uncertainty—the risk that the AI policy overfits to the simulated environment and fails when deployed to the physical RAN.

To mitigate model mismatch and concept drift over time, the DT must implement mechanisms for periodic re-calibration. This involves continuously updating its internal environmental models (e.g., adjusting the dielectric properties of building materials in the ray-tracing engine) using real-world crowdsourced data and the aforementioned E2 KPIs. Furthermore, because wireless channels are fundamentally non-stationary (e.g., due to seasonal foliage changes or new urban construction), the DRL agent employs continuous online fine-tuning using a low learning rate and its experience replay buffer to seamlessly adapt to concept drift without requiring a complete offline retraining cycle.

### 8.3. Practical RIS Constraints: Quantization and Amplitude-Phase Coupling

While the vast majority of existing system-level RIS literature relies exclusively on the ideal unit-modulus assumption, our primary beamforming optimization (Section 4) acknowledges this limitation. Under the ideal assumption, the amplitude reflection coefficient is maximized and decoupled from the phase shift ($\beta_{r,q}=1$, ∀r,q).

In physical meta-surfaces, however, the amplitude response is intrinsically coupled to the applied phase shift due to the equivalent circuit characteristics of the semiconductor elements. Maximizing the phase excursion typically results in a minimum reflection amplitude, causing signal attenuation [31]. Furthermore, hardware limitations restrict phase shifts to discrete, quantized values (e.g., 2-bit or 3-bit resolution).

To rigorously quantify the impact of these physical limitations on the reported network gains, we conduct an analytical bounding analysis. We model the practical amplitude-phase coupling using the established empirical function [31]

$$\beta_{r,q}\left(\theta_{r,q}\right)=\left(1-\beta_{\min}\right){\left(\frac{\sin(\theta_{r,q}-\phi )+1}{2}\right)}^{\alpha }+\beta_{\min}$$

where $\beta_{\min}$ is the minimum amplitude at the worst-case phase shift, ϕ is the phase shift at which the minimum amplitude occurs, and α controls the steepness of the attenuation curve. For typical hardware operating at our simulated frequencies, we set the physical parameters to $\beta_{\min}=0.2$, ϕ=0.43π, and α=1.6.

We applied this coupling model, combined with a 2-bit discrete phase quantization constraint (allowing only $\Theta_{r,q}\in \left\{0,\pi /2,\pi ,3\pi /2\right\}$), as a parametric sweep over the optimized phase matrices ($\Theta^{*}$) generated by the digital twin in our $1\;{\mathrm{km}}^{2}$ dense urban topology.

Bounding Analysis Results: The combined effect of 2-bit quantization and amplitude-phase coupling introduces a mean per-element reflection loss of approximately 3.8dB relative to the ideal unit-modulus case. When cascaded through the network, this translates to the following performance bounds:

- Spectral Efficiency (SE): The mean SE decreases from the ideal simulated 4.6bits/s/Hz to a bounded realistic value of ≈4.1bits/s/Hz. Despite this hardware penalty, the proposed hybrid framework still yields a 28% capacity improvement over the 3.2bits/s/Hz legacy baseline.
- Coverage Gains: The signal attenuation diminishes the penetration capability in deep NLOS urban canyons. The total reduction in coverage holes shifts from an ideal 84% to a realistic lower bound of 71%.

This bounding analysis confirms that while hardware non-idealities measurably degrade the absolute theoretical peaks of RIS-assisted CF-mMIMO, the macro-diversity and passive beamforming gains remain vastly superior to traditional collocated 5G architectures.

### 8.4. CAPEX Optimization: Passive RIS vs. Active Densification

From an operator’s perspective, the transition to B5G/6G architectures is often hindered by prohibitive CAPEX associated with active antenna densification. Traditional deployment strategies rely on “brute-force” active densification, requiring extensive fiber-optic fronthaul expansion and dedicated RF chains for every new coverage node. The proposed framework demonstrates that integrating passive RIS panels as extensions of the distributed active array can recover up to 40% of viable coverage in NLOS urban canyons without the need for additional active hardware. Furthermore, by leveraging the O-RAN 7.2x functional split, the framework optimizes the utilization of existing legacy 5G infrastructure, significantly reducing the cost-per-bit while achieving an 84% reduction in coverage holes. This shift from active-only to hybrid active–passive architectures provides a sustainable and cost-effective migration path for mobile network operators.

### 8.5. Research Limitations and Open Challenges

While the proposed framework and its simulation results present a compelling case for the adoption of RIS-assisted CF-mMIMO architectures, several important assumptions and validation boundaries must be explicitly acknowledged to characterize the scope and generalizability of the present findings.

Ideal Unit-Modulus RIS Assumption. The beamforming optimization in Section 4 adopts the ideal unit-modulus constraint ($\beta_{r,q}=1,\;\forall \;r,q$), which assumes that every RIS element reflects incident signals with unity amplitude regardless of the applied phase shift. In reality, practical meta-surface hardware exhibits an inherent amplitude–phase coupling: maximizing the phase excursion tends to reduce the reflection coefficient, a phenomenon whose physical origins and modeling are detailed in [10]. Although Section 8.3 demonstrates that 2-bit quantized phase resolution incurs only a manageable ≈1.5 dB penalty, a full hardware-aware formulation incorporating phase-dependent amplitude loss and inter-element mutual coupling is beyond the scope of this initial framework and is reserved as immediate future work.

Simulation-Only Validation. All performance results presented in Section 7 are derived exclusively from Monte Carlo simulations conducted in Atoll and MATLAB, calibrated against the 3GPP TR 38.901, COST-231, and ITU-R/ETSI RIS propagation models. Although the simulation inputs are drawn from real operator measurement campaigns (drive-test and walk-test traces) and the N≥1000 Monte Carlo realizations provide 95% statistical confidence, no OTA testbed or hardware-in-the-loop (HIL) validation has been performed. Experimental verification using software-defined radio (SDR) platforms or commercial O-RAN deployments with physical RIS panels is an essential next step to confirm algorithm viability in the presence of real hardware impairments such as phase noise, amplifier non-linearities, and eCPRI synchronization jitter.

Pilot Contamination Modeling. The dynamic pilot assignment implemented in Layer 3 of the framework adopts a simplified traffic-aware clustering approach. In practice, pilot contamination in ultra-dense CF-mMIMO deployments—with up to 500 UEs/km^2^ sharing the same time-frequency resources across hundreds of distributed APs—is a considerably more complex problem. Advanced mitigation strategies such as graph-coloring-based pilot reuse, statistical channel aging models, and covariance-aided estimation for mobile users are not fully incorporated in the current framework. The reported 30% aggregate interference reduction should therefore be interpreted as achievable under the idealized pilot assignment conditions of the simulation and may be lower in deployments with high user mobility or significant channel non-stationarity.

Sensitivity Bounds to Hardware Impairments. While the simulated framework demonstrates a 45% gain in spectral efficiency, these results assume ideal hardware transceivers. In practical O-RAN deployments, the achievable capacity is strictly bounded by hardware impairments—specifically power amplifier (PA) non-linearities, local oscillator phase noise, and fronthaul synchronization jitter [32,33]. To contextualize our simulation claims, we establish the following quantitative sensitivity bounds:

1. PA Non-Linearity and Phase Noise (EVM Bound): The aggregate effect of transceiver impairments can be modeled using the error vector magnitude (EVM), denoted as κ. Under this model, the effective SINR for user *k*, $\gamma_{k}^{\mathrm{eff}}$, is degraded proportionally to the ideal simulated SINR, $\gamma_{k}^{\mathrm{ideal}}$:

$$\gamma_{k}^{\mathrm{eff}}\approx \frac{\gamma_{k}^{\mathrm{ideal}}}{1+\kappa^{2}\cdot \gamma_{k}^{\mathrm{ideal}}}$$

In standard sub-6 GHz deployments, commercial active antenna units maintain an EVM of κ≈0.08 (8%), which is sufficient for 64-QAM. However, as $\gamma_{k}^{\mathrm{ideal}}\to \infty$, the effective SINR hits a hard hardware ceiling at $1/\kappa^{2}$. For κ=0.08, the maximum achievable SINR is strictly bounded at ≈21.9dB. Consequently, the highest SINR values reported in the 95th percentile of our empirical CDFs (Figure 3a) would be truncated in a physical deployment unless high-linearity, pre-distorted PAs (achieving κ<0.03) are utilized.

2. eCPRI Synchronization Jitter at mmWave: CF-mMIMO relies on coherent joint transmission across geographically distributed access points (APs). This requires extremely tight phase synchronization over the eCPRI fronthaul. A timing synchronization error (jitter) of Δτ seconds results in a carrier phase error of $\Delta \phi =2\pi f_{c}\Delta \tau$, where $f_{c}$ is the carrier frequency.

To prevent destructive interference and maintain a beamforming gain penalty of less than 1dB, the phase error variance must satisfy $\sigma_{\phi }\leq \pi /6$ radians [34]. This imposes a strict timing error bound:

$$\Delta \tau \leq \frac{1}{12f_{c}}$$

For our 3.5GHz macro-tier, the synchronization bound is Δτ≤23.8ps, which is highly challenging but theoretically achievable with state-of-the-art PTP Class D boundary clocks. However, for the 28GHz mmWave tier, the bound drops to a prohibitive Δτ≤2.9ps. Standard Ethernet-based O-RAN fronthaul networks currently exhibit jitters in the nanosecond range, meaning that without dedicated phase-synchronization overlays or shifting to non-coherent transmission schemes, the simulated coherent mmWave gains will break down entirely in field deployments.

## 9. Limitations and Future Research Directions

This work validates the functional integration of DTs with MADRL for O-RAN compliant CF-mMIMO RIS optimization. However, several limiting factors remain regarding physical model fidelity and O-RAN system dynamics, which provide clear directions for future work.

Regarding physical modeling, our simplified channel assumptions represent an optimistic upper bound on performance. Standard hardware impairments (HWI) at the CP-CF-APs and the RIS controller can significantly degrade beamforming gain and robustness compared to ideal modeling [35]. Similarly, the practical efficacy of dense RIS deployments is bounded by phase-shift quantization errors (limited resolution bits) and strong mutual coupling among adjacent RIS elements [36]. While mutual coupling significantly complicates the passive beamformer design matrix, phase quantization imposes strict performance floors that cannot be exceeded by multi-agent policies alone.

Regarding O-RAN architectural dynamics, finite fronthaul capacity remains a significant practical bottleneck in CF-mMIMO. While standard fiber or eCPRI fronthaul capacity easily handles standard RIS controller traffic, the aggregated data traffic required for coherent CF-mMIMO joint processing is massive. The optimal policy must incorporate fronthaul load balancing and traffic-aware scheduling within its state vector to avoid congestion and overall user performance degradation. Furthermore, dynamic UE mobility and bursty traffic dynamics will necessitate state vectors $s_{t}$ that incorporate temporal history (e.g., via LSTM or Transformer layers) or attention mechanisms to effectively track evolving network patterns. Future work will extend this multi-agent DT architecture to advanced apertures and architectures, such as Star-RIS [14] or intelligent omnisurfaces [15], optimizing omnidirectional coverage patterns for mobile users within dense DT environments.

## 10. Conclusions

The transition from legacy 5G to 6G requires a structural shift from cell-centric to user-centric architectures. This paper presented a scalable, hybrid active–passive CF-mMIMO framework that empowers network operators to resolve severe cell-edge interference and NLOS coverage gaps without incurring the prohibitive CAPEX of dense active fiber deployments.

By integrating a DT within the O-RAN 7.2x architecture and utilizing MADRL alongside FP, the framework achieves autonomous, real-time beamforming optimization. Extensive simulations over a dense urban topology demonstrate that this approach yields a 45% increase in spectral efficiency, a 30% reduction in uplink interference, and an 84% reduction in coverage holes.

Despite these significant simulated gains, critical challenges remain unsolved for physical deployment. The theoretical performance presented is fundamentally bounded by practical hardware impairments, particularly RIS amplitude-phase coupling, finite quantization errors, and the strict eCPRI synchronization jitter limits required for coherent mmWave transmission.

Consequently, the clear next steps for this research involve transitioning from simulation to physical OTA hardware-in-the-loop testing. Future work will focus on integrating robust, hardware-impairment-aware beamforming models and exploring multi-band spectrum aggregation to validate this architectural blueprint for intelligent, ultra-efficient 6G networks.

## Figures and Tables

**Table 1:** Comparison of features between the paper baseline [1] and this work.

Component	Baseline [1]	This Work
4-Layer Planning Framework	Basic Logic	Extended (O-RAN 7.2×)
RIS-Assisted Passive Beamforming	Not Addressed	Full Optimization Layer
Digital Twin/MADRL Feedback Loop	Not Addressed	Real-Time Adaptive Loop
Simulation Scope	Stochastic Models	Dense Urban Monte Carlo
Algorithmic Complexity Paradigm	Static Offline Optimization	Real-Time DT Inference ($O(L_{n}\cdot N_{n})$)

**Table 2:** Notation and Symbol Glossary.

Symbol	Dimension/Domain	Definition
L,K,R	$Z^{+}$	Total number of active APs, UEs, and RIS panels.
N,Q	$Z^{+}$	Number of antennas per AP and reflecting elements per RIS.
*M*	$Z^{+}$	Total number of distributed transmit antennas (M=L×N).
$w_{k}$	$C^{M\times 1}$	Network-wide active beamforming (precoding) vector for UE *k*.
$\Theta_{r}$	$C^{Q\times Q}$	Diagonal phase-shift reflection matrix for RIS panel *r*.
$\Theta_{r,q}$	[0,2π)	Continuous phase-shift applied by the *q*-th element of RIS *r*.
$h_{k}$	$C^{M\times 1}$	Global effective cascaded channel vector from all APs to UE *k*.
$d_{l,k}$	$C^{N\times 1}$	Direct channel vector from AP *l* to UE *k*.
$G_{l,r}$	$C^{Q\times N}$	Channel matrix from AP *l* to RIS panel *r*.
$f_{r,k}$	$C^{Q\times 1}$	Channel vector from RIS panel *r* to UE *k*.
$\gamma_{k}$	$R^{+}$	Effective downlink SINR for UE *k*.

**Table 3:** End-to-end DT control-loop latency budget for the Near-RT RIC architecture. Values are estimated from O-RAN Alliance evolved common public radio interface (eCPRI) specifications [18] and published empirical O-RAN testbed measurements [26].

Processing Stage	Latency (ms)	Notes
E2 measurement collection (O-DU → Near-RT RIC)	5–15	eCPRI + E2 framing; periodic report interval
Fronthaul transport (O-DU → Near-RT RIC)	2–8	Ethernet/IP latency, queue depth
DT state update & channel reconstruction	5–20	Ray-tracing re-sync; GPU-accelerated
MADRL policy inference (compressed state, GPU)	<1	Feed-forward pass through actor networks
Policy deployment via E2/A1 interface	5–10	JSON encoding + E2 transport
O-RU/AP beamforming reconfiguration	1–8	Hardware update latency
Total round-trip (estimated)	18–62 ms	Within Near-RT RIC window: 10–100 ms

**Table 4:** Key Simulation Parameters for Dense Urban Environment.

Parameter	Value/Configuration	Operational Context
Simulated Area	$1\;{\mathrm{km}}^{2}$ Dense Urban Topology	High-rise buildings (5–50 m), 20 m street widths.
Spectrum Bands	3.5 GHz (Sub-6) & 28 GHz (mmWave)	Targeted dual-band B5G deployment.
Bandwidth	100 MHz (3.5 GHz)/400 MHz (28 GHz)	High-capacity payload simulation.
Network Scale (L,R)	L=12 distributed APs, R=6 RIS panels	Cooperative active–passive topology.
Active AP Antenna Arrays	8×8 URA (3.5 GHz)/16×16 URA (28 GHz)	Distributed spatial multiplexing elements.
Passive RIS Elements	256 elements per panel (16×16)	Meta-surface phase shifters for NLOS reflection.
AP Transmit Power	30 dBm per Access Point	Standard micro-cell equivalent power limits.
Propagation Models	3GPP TR 38.901 (UMa/UMi), COST-231	Multipath and diffraction modeling.
User Density	Up to 500 UEs/km^2^	Stress-testing capacity and pilot contamination.
Signal Processing	MMSE Channel Est., ZF/MMSE Precoding	Optimized via FP.
Monte Carlo Realizations	≥1000 iterations	95% confidence interval (±0.5–1 dB) statistical validity.

**Table 5:** Performance benchmarking of CF-mMIMO architectures and computational complexity analysis. Complexity is expressed in terms of dominant floating-point operations (FLOPs) per AO iteration (Algorithm 1) for *L* APs, *N* antennas/AP, *K* UEs, *Q* RIS elements, and *R* RIS panels. Reference parameters: L=12, N=256 (mmWave), K=20, Q=256, R=6. The ablation row (“Hybrid RIS + FP-AO, no DT”) isolates the contribution of static offline optimization from that of the DT real-time adaptation loop. Note: Algorithmic FLOPs shown here are strictly decoupled from the ≈15% operational wall-clock latency overhead discussed in Section 6.4, which relates solely to MADRL inference time.

Architecture/Framework	Avg. Spectral Eff.	Uplink Interf.	NLOS Coverage	Dominant Complexity
Legacy 5G collocated mMIMO (baseline)	3.2 bits/s/Hz	Baseline	Minimal (active only)	$O(K^{3})$ per cell
Fully centralized CF-mMIMO	3.8 bits/s/Hz	Moderate (−10%)	Minimal (active only)	$O(M^{3}+KM^{2})$
Distributed CF-mMIMO (no RIS)	4.2 bits/s/Hz	High (−22%)	Minimal (active only)	$O(LN^{3}+KLN^{2})$
Hybrid RIS + FP-AO, static (no DT) [ablation]	4.4 bits/s/Hz (+38%)	High (−26%)	+30% via passive RIS	$O(M^{3}+KM^{2}+RQ^{2})$
Proposed (Hybrid RIS + DT)	4.6 bits/s/Hz (+45%)	High (−30%)	+40% via passive RIS	$O(M^{3}+KM^{2}+RQ^{2})$

**Table 6:** Comparison of the Proposed Framework with State-of-the-Art RIS-Assisted CF-mMIMO Literature.

Framework/Reference	Year	Optimization Method	Real-Time Loop	O-RAN Compliant	Reported Gain (vs. Baseline)
Zhang et al. [9]	2021	WMMSE/SCA (Static)	No (Offline)	No	≈35% SE gain (Theoretical)
Liu et al. [28]	2023	Single-Agent DDPG	DRL only	No	≈30% SE gain (Simulated)
Wu et al. [29]	2024	AO + Federated Learning	Yes (DT)	No	≈42% SE gain (Simulated)
Proposed Framework	2026	FP-AO + MADRL	Yes (DT + MADDPG)	Yes (7.2x Split)	45% SE gain, 84% coverage recovery

## Data Availability

The data presented in this study are available on request from the corresponding author.

## Abbreviations

The following abbreviations are used in this manuscript:

AO	Alternating Optimization
AP	Access Point
B5G	Beyond Fifth-Generation
CF-mMIMO	Cell-Free Massive Multiple-Input Multiple-Output
CAPEX	Capital Expenditure
CPU	Central Processing Unit
CSI	Channel State Information
DRL	Deep Reinforcement Learning
DT	Digital Twin
E2	O-RAN E2 Interface (near-RT RIC to E2 Node)
FP	Fractional Programming
HPBW	Half-Power Beamwidth
ICI	Inter-Cell Interference
ITU-R	International Telecommunication Union–Radiocommunication Sector
KKT	Karush–Kuhn–Tucker
KPI	Key Performance Indicator
LDT	Lagrangian Dual Transform
MADRL	Multi-Agent Deep Reinforcement Learning
MADDPG	Multi-Agent Deep Deterministic Policy Gradient
MDP	Markov Decision Process
MMSE	Minimum Mean Square Error
mmWave	Millimeter-Wave
mMIMO	Massive Multiple-Input Multiple-Output
NLOS	Non-Line-of-Sight
NSA/SA	Non-Stand-Alone/Stand-Alone
O-DU	Open Distributed Unit
O-RAN	Open Radio Access Network
O-RU	Open Radio Unit
QCQP	Quadratically Constrained Quadratic Program
QoS	Quality of Service
QT	Quadratic Transform
RAN	Radio Access Network
RIS	Reconfigurable Intelligent Surface
RSRP	Reference Signal Received Power
SAC	Soft Actor–Critic
SCA	Successive Convex Approximation
SINR	Signal-to-Interference-plus-Noise Ratio
SLL	Sidelobe Level
SRM	Sum-Rate Maximization
UE	User Equipment
URA	Uniform Rectangular Array
ZF	Zero-Forcing
6G	Sixth-Generation
